# Identifying PRKAG2 syndrome—a rare cause of wolff-Parkinson-white syndrome and left ventricular hypertrophy: a case report

**DOI:** 10.1093/ehjcr/ytaf310

**Published:** 2025-06-28

**Authors:** Ji-hun Jang, Yong-Soo Baek, Woo-Ri Jang, Dae-Young Kim, Sung-Hee Shin

**Affiliations:** Department of Cardiology, Inha University Hospital Cardiovascular Centre, Inha University Hospital, 27 Inhang-ro, Jung-gu, Incheon 400-711, Republic of Korea; Department of Cardiology, Inha University Hospital Cardiovascular Centre, Inha University Hospital, 27 Inhang-ro, Jung-gu, Incheon 400-711, Republic of Korea; Department of Laboratory Medicine, College of Medicine, Inha University, 100 Inha-ro, Michuholgu, Incheon 22212, Republic of Korea; Department of Cardiology, Inha University Hospital Cardiovascular Centre, Inha University Hospital, 27 Inhang-ro, Jung-gu, Incheon 400-711, Republic of Korea; Department of Cardiology, Inha University Hospital Cardiovascular Centre, Inha University Hospital, 27 Inhang-ro, Jung-gu, Incheon 400-711, Republic of Korea

**Keywords:** Wolff-Parkinson-White syndrome, Left ventricular hypertrophy, PRKAG2 mutation, Sudden cardiac death, Case report

## Abstract

**Background:**

In young patients presenting with Wolff-Parkinson-White syndrome and left ventricular hypertrophy (LVH), a genetic aetiology should be considered, particularly when cardiovascular risk factors such as hypertension or valvular heart disease are not present, or when there is a suggestive family history of cardiomyopathy. PRKAG2 syndrome is a rare genetic disorder characterized by cardiac glycogen storage, ventricular pre-excitation, and hypertrophy, often mimicking hypertrophic cardiomyopathy (HCM).

**Case Summary:**

A 22-year-old male with a history of WPW syndrome presented with recurrent palpitations and wide QRS tachycardia. Post-cardioversion electrocardiography (ECG) revealed persistent pre-excitation, and transthoracic echocardiography confirmed LVH with preserved systolic function. Cardiac magnetic resonance imaging demonstrated normal myocardial mass without late gadolinium enhancement. Genetic testing, prompted by a family history of sudden cardiac death (SCD), using a targeted panel sequencing including sarcomere protein gene mutations and other cardiomyopathy-related genes, identified a heterozygous PRKAG2 mutation. Given the high-risk of SCD, implantable cardioverter-defibrillator placement was recommended but declined. The patient subsequently experienced a fatal cardiac arrest 8 days after the last clinic visit.

**Discussion:**

This case highlights the importance of genetic evaluation in young patients with unexplained arrhythmias and hypertrophy. PRKAG2 mutations, often overlooked in standard HCM panels, can lead to misdiagnosis and inadequate risk stratification. Clinicians should maintain a high index of suspicion for PRKAG2 syndrome, particularly in patients with conduction abnormalities, ventricular arrhythmias, and LVH. Early identification using genetic tests, risk assessment, and family screening are crucial to preventing adverse outcomes, including SCD.

## Graphical Abstract

**Graphical Abstract:**
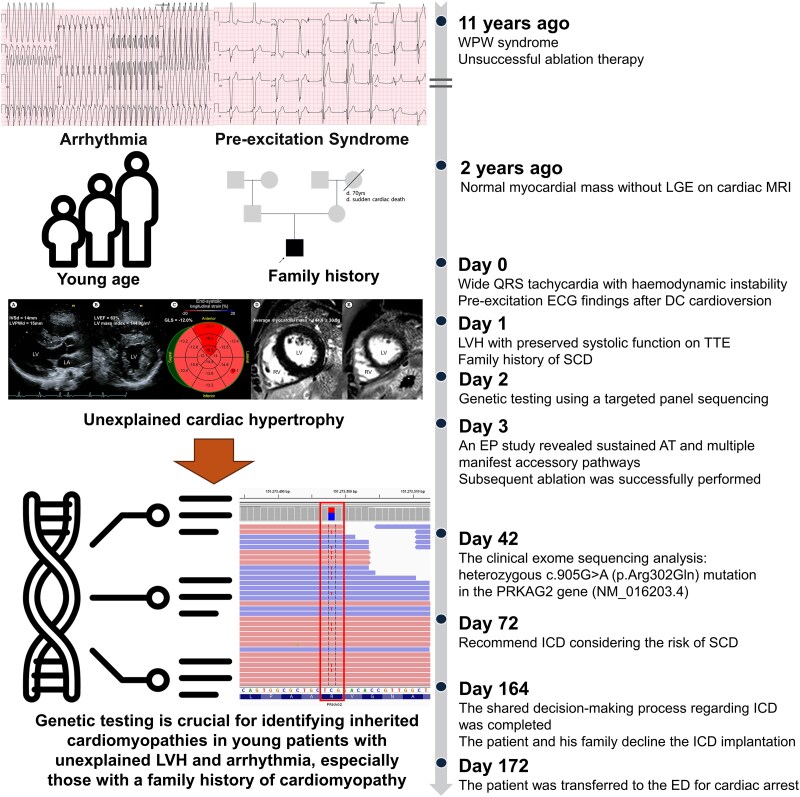

Learning points

- Genetic testing is essential for identifying mutations in young patients with unexplained arrhythmias and hypertrophy, guiding accurate diagnosis and treatment.
- Comprehensive risk assessment and family screening for patients with PRKAG2 mutations are crucial to reduce the risk of SCD and identify other genetic cardiomyopathies within affected families.
- Further genetic testing should be considered for PRKAG2 mutations and other possible causes, even if sarcomere gene mutation analysis results are negative in these conditions.

## Introduction

Differential diagnosis of left ventricular hypertrophy (LVH) is often challenging, particularly when it is ‘unexplained’. While LVH is traditionally associated with physiological consequences of increased afterload due to conditions like hypertension and valvular heart disease, it can also manifest as a consequence of various cardiomyopathies, including hypertrophic or infiltrative cardiomyopathies.^1,2^ In individuals presenting with LVH, especially in the absence of overt risk factors and accompanied by specific arrhythmias, the potential for inherited aetiologies warrants significant attention.^3^ Herein, we detail the presentation of a young male who manifested with Wolff-Parkinson-White (WPW) syndrome and LVH, ultimately diagnosed with PRKAG2 syndrome.

## Case

A 22-year-old male presented to the emergency department (ED) with palpitations. He had been diagnosed with WPW syndrome 11 years ago and had experienced recurrent episodes of palpitations. Despite undergoing ablation therapy for an accessory pathway at an outside hospital, the procedure was unsuccessful. The patient did not maintain regular follow-up visits. At the time of initial diagnosis, transthoracic echocardiography (TTE) demonstrated mildly increased left ventricular (LV) wall thickness, with interventricular septal thickening (11 mm) and posterior wall thickness (13 mm). Left ventricular ejection fraction (LVEF) was 75%.

**Figure 1:**
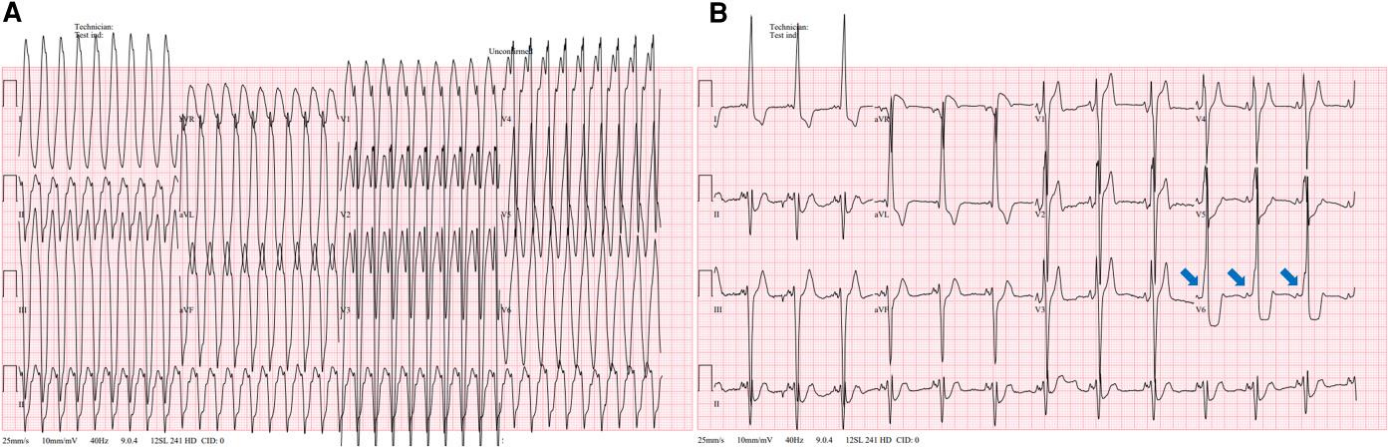
(*A*) Initial ECG showing a wide complex tachycardia with QRS complexes measuring 164 ms and a rate of 276 beats per minute, indicating ventricular tachycardia. The axis is leftward with a predominantly negative deflection in the inferior leads. (*B*) Post-cardioversion ECG illustrates sinus rhythm with a heart rate of 85 beats per minute, a short PR interval of 90 ms, and prominent pre-excitation delta waves in lead V6 (arrows), indicative of WPW syndrome. ECG, electrocardiography; ED, emergency department; WPW, Wolff-Parkinson-White.

**Figure 2:**
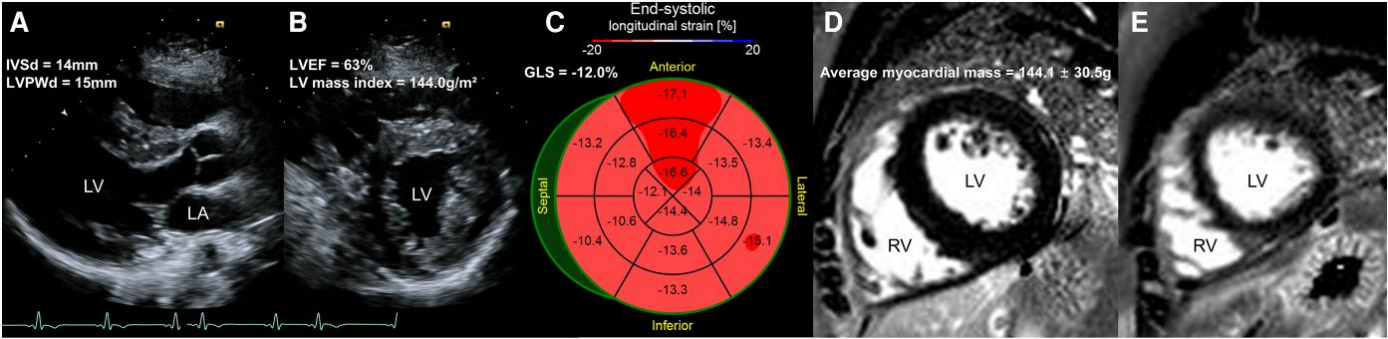
(*A*, *B*) Transthoracic echocardiography demonstrates left ventricular hypertrophy, with interventricular septal thickening (14 mm) and increased posterior wall thickness (15 mm), consistent with concentric hypertrophy (*C*) global longitudinal strain: bull's eye plot. (*D*, *E*) Cardiac magnetic resonance image reveals normal myocardial thickening and left ventricle mass with no late gadolinium enhancement. GLS, global longitudinal strain; IVSd, interventricular septal diameter; LA, left atrium; LV, left ventricle; LVEF, left ventricular ejection fraction; LVPWd, left ventricular posterior wall diameter; RV, right ventricle.

Upon arrival at the ED, the patient appeared visibly distressed, with signs of haemodynamic instability. On physical examination, the patient was tachycardic, anxious, and diaphoretic. Cardiac auscultation revealed a rapid but regular rhythm with no murmurs, rubs, or gallops. Pulmonary auscultation was clear without rales or wheezing. No peripheral oedema or jugular venous distension was noted. Initial vital signs recorded a heart rate of 276 beats per minute and blood pressure of 90/62 mmHg. A 12-lead electrocardiogram (ECG) showed a regular, wide QRS tachycardia without clearly discernible *P* waves, raising a suspicion for antidromic atrioventricular re-entrant tachycardia via an accessory pathway—consistent with the patient’s known WPW syndrome—or ventricular tachycardia (*Figure 1A*). Synchronized DC cardioversion at 100J was successfully performed, achieving sinus rhythm conversion. Post-cardioversion ECG exhibited a prominent pre-excitation delta wave, suggesting accessory pathway conduction (*Figure 1B*). Initial laboratory studies indicated the following results: Creatine kinase, 85 U/L (reference range: 20–200 U/L); creatine kinase-myocardial band, 5.1 ng/mL (reference range: 0.0–0.5 ng/mL; elevated); and troponin I, 0.12 ng/mL (reference range: < 0.02 ng/mL; elevated). The *n*-terminal pro-B-type natriuretic peptide (NT-proBNP) level was 192 pg/mL (reference range: 0–125 pg/mL; mildly elevated). Comprehensive TTE demonstrated concentric LVH, with the most prominent thickening in the mid-interventricular septum (maximum thickness: 16 mm). LV systolic function was preserved, with an LVEF of 63%, and the LV mass index was elevated at 144.0 g/m², indicative of hypertrophy (*Figure 2A and B*; Supplementary material online, *Videos S1*–*S3*). Global longitudinal strain was reduced to −12.0% (*Figure 2C*). The patient had no history of hypertension, and his blood pressure remained within the normal range throughout the evaluation.

Considering the pre-excitation ECG findings and LVH on TTE, further investigation into potential causes such as infiltrative cardiomyopathies or genetic cardiac disorders, including hypertrophic cardiomyopathy (HCM), was warranted. A review of his cardiac magnetic resonance imaging from 2 years prior (*Figure 2D and E*; Supplementary material online, *Video S4*) indicated no late gadolinium enhancement, with an average myocardial mass of 144.1 ± 30.5 g (reference range: 96–200 g).^4^

**Figure 3:**
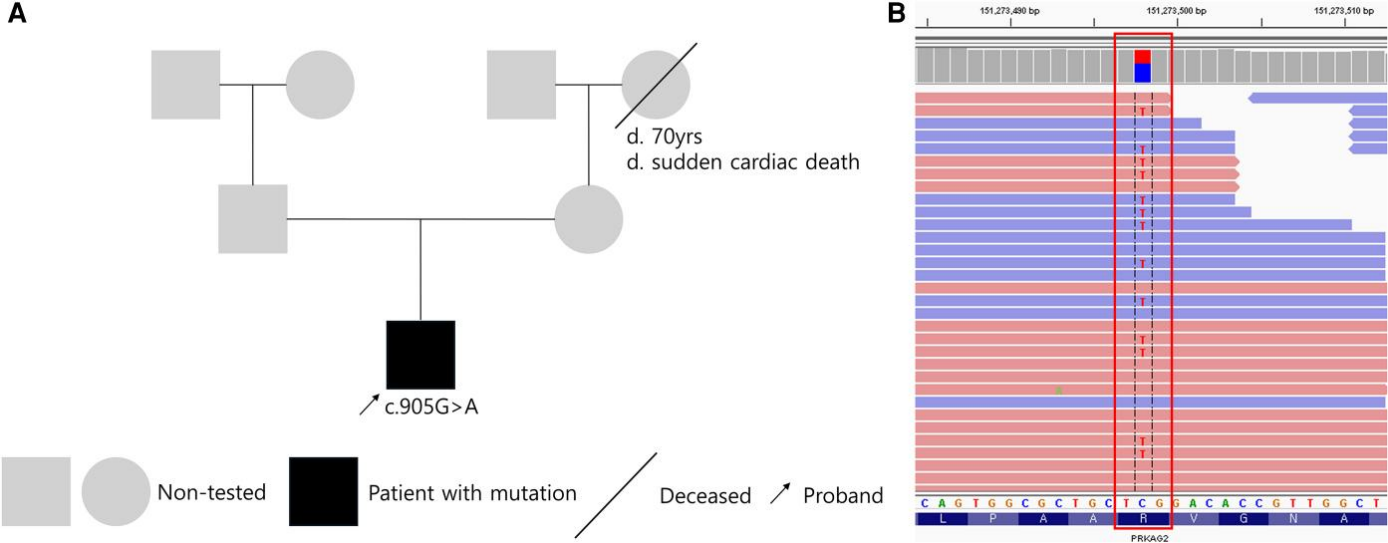
(*A*) Pedigree structure of the family. Squares indicate males, and circles, females. Affected individuals are represented by filled symbols. Grey symbols indicate non-tested individuals. A solid black arrow indicates the proband. Slants denote dead individuals. (*B*) Integrative Genomics Viewer snapshot showing the pathogenic variant of PRKAG2 gene in the proband (NM_016203.4:c.905G > A).

Given his family history, notably a grandmother who died suddenly of presumed cardiac origin, the genetic investigation was prioritized (*Figure 3A*). Testing for Fabry disease and next-generation sequencing targeting sarcomere protein gene mutations showed negative results. However, given his distinctive clinical features and family history, further genetic testing was conducted to explore other genetic and metabolic cardiomyopathies that can mimicHCM. The clinical exome sequencing analysis of the proband's germline revealed a heterozygous c.905G > A (p.Arg302Gln) mutation in the PRKAG2 gene (NM_016203.4), confirming the diagnosis of glycogen storage cardiomyopathy (*Figure 3B*). According to the ACMG guidelines,^5^ the p.Arg302Gln variant was classified as pathogenic and has been recurrently reported in several patients.^6,7^ During hospitalisation, the patient underwent a repeat electrophysiological study, which revealed sustained atrial tachycardia and multiple manifest accessory pathways. Successful ablation was achieved using a 3D mapping system, and no arrhythmia was inducible post-procedure. He was discharged on bisoprolol 5 mg daily, later tapered to 2.5 mg during follow-up. Considering the risk of sudden cardiac death (SCD) in patients with PRKAG2 mutations and the patient’s ventricular tachyarrhythmia, we recommended implantation of an implantable cardioverter-defibrillator (ICD) following detailed discussion with the patient and his relatives, and advised family screening for PRKAG2 mutations.

Despite the recommendation, the patient and his family hesitated about ICD implantation and refused familial screening. Tragically, the patient was transferred to the ED for cardiac arrest 8 days after the last outpatient clinic visit. His initial rhythm was asystole, and resuscitation efforts were unsuccessful. The patient was pronounced deceased.

## Discussion

PRKAG2 syndrome is an autosomal dominant disorder first mapped to the locus 7q36 in 1991, with the responsible gene identified in 2001.^3,6,7^ The syndrome results from mutations in PRKAG2, which encodes the γ2 regulatory subunit of adenosine monophosphate-activated protein kinase (AMPK). AMPK is a crucial enzyme in cellular ATP metabolism, regulating glucose uptake and glycolysis. PRKAG2 mutations induce structural changes in AMPK, leading to impaired glucose utilisation in myocytes and subsequent glycogen storage cardiomyopathy. This leads to glycogen accumulation, ventricular pre-excitation, hypertrophy, and increased arrhythmic risk.^6–8^ While PRKAG2 syndrome phenotypically overlaps with HCM, its pathophysiology and clinical course differ significantly, necessitating specific diagnostic considerations.^7^

The distinguishing features of PRKAG2 syndrome include early-onset WPW syndrome, progressive conduction disease, and arrhythmias, including atrial fibrillation and ventricular tachycardia.^6,7^ Unlike sarcomeric HCM, PRKAG2 cardiomyopathy is not associated with myocyte disarray but rather with glycogen storage, which affects electrophysiological properties. This leads to unique ECG findings such as a short PR interval, right bundle branch block, and frequent pre-excitation patterns.

Comprehensive genetic testing, including PRKAG2 mutation analysis, is crucial for differentiating PRKAG2 syndrome from sarcomeric HCM and other metabolic cardiomyopathies, ensuring appropriate risk stratification and early intervention. Among PRKAG2-related mutations, p.Arg302Gln is the most frequently reported pathogenic variant strongly associated with ventricular pre-excitation, particularly WPW syndrome. This variant exhibits considerable phenotypic variability, ranging from asymptomatic carriers to individuals with severe HCM and progressive conduction disease requiring pacemaker implantation.^6,9^ Given these clinical implications, early genetic identification of this variant is crucial for timely diagnosis, risk stratification, and implementation of preventive strategies. Also, our case highlights the limitations of standard HCM genetic panels, which primarily screen for sarcomeric gene mutations and often exclude PRKAG2.^3,8^ This can result in missed diagnoses and inadequate risk stratification, underscoring the necessity of expanded genetic analysis in select patients.

SCD is a well-recognized complication of PRKAG2 syndrome, occurring independently of hypertrophy severity. The underlying mechanisms include malignant ventricular arrhythmias and progressive conduction disease. Current guidelines recommend ICD placement for primary prevention in high-risk individuals; however, risk stratification remains challenging due to the heterogeneity of phenotypic expression.^8^ The reported incidence of SCD in PRKAG2 syndrome varies, but it has been observed in up to 8.7% of patients, as shown in an observational study of 171 individuals.^6^ Moreover, another study reported that patients with the same genetic mutation as our case exhibited up to a 10-fold higher SCD event rate than other subsets.^7^ In our cohort, the decision for prophylactic ICD implantation was based on the patient's phenotype and family history of SCD, particularly in symptomatic patients with ventricular tachycardia.

Our case illustrates the tragic consequences of ICD refusal, emphasising the need for patient education and shared decision-making in managing hereditary cardiomyopathies. Additionally, this case highlights the necessity of family screening and genetic counselling to enable early diagnosis and appropriate risk management for at-risk relatives.

In conclusion, clinicians should maintain a high degree of suspicion for PRKAG2 syndrome in patients presenting with WPW syndrome and unexplained LVH, particularly when accompanied by a family history of SCD. Expanding genetic testing beyond sarcomeric gene panels is essential to identify rare but clinically significant conditions, optimize risk management, and prevent adverse outcomes such as SCD.

## Lead author biography


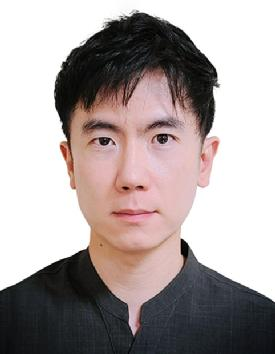


Dr Ji-hun Jang is an assistant professor of cardiology at Inha University Hospital, specialising in heart failure. He completed his residency and cardiology fellowship at Inha University Hospital, followed by a clinical research assistant professorship at Severance Hospital, Yonsei University. His interests include medical therapy for heart failure, advanced heart failure management, mechanical circulatory support, and cardiomyopathies.

## Supplementary Material

ytaf310_Supplementary_Data

## Data Availability

The data of this case report are available from the corresponding author upon reasonable request.
